# Sirt3-Mediated Autophagy Contributes to Resveratrol-Induced Protection against ER Stress in HT22 Cells

**DOI:** 10.3389/fnins.2018.00116

**Published:** 2018-02-27

**Authors:** Wen-Jun Yan, Ruo-Bin Liu, Ling-Kai Wang, Ya-Bing Ma, Shao-Li Ding, Fei Deng, Zhong-Yuan Hu, Da-Bin Wang

**Affiliations:** Department of Anesthesiology, Gansu Provincial Hospital, Lanzhou, China

**Keywords:** resveratrol, autophagy, ER stress, Sirt3, HT22 cells

## Abstract

Endoplasmic reticulum (ER) stress occurring in stringent conditions is critically involved in neuronal survival and death. Resveratrol is a non-flavonoid polyphenol that has neuroprotective effects against many neurological disorders. Here, we investigated the potential protective effects of resveratrol in an *in vitro* ER stress model mimicked by tunicamycin (TM) treatment in neuronal HT22 cells. We found that TM dose-dependently decreased cell viability and increased apoptosis, which were both significantly attenuated by resveratrol treatment. Resveratrol markedly reduced the expression or activation of ER stress-associated factors, including GRP78, CHOP, and caspase-12. The results of immunocytochemistry and western blot showed that resveratrol promoted autophagy in TM-treated cells, as evidenced by increased LC3II puncta number, bcelin1 expression and LC3II/LC3I ratio. Pretreatment with the autophagy inhibitor chloroquine could reduce the protective effects of resveratrol. In addition, the expression of Sirt3 protein and its downstream enzyme activities were significantly increased in resveratrol-treated HT22 cells. To confirm the involvement of Sirt3-mediated mechanisms, siRNA transfection was used to knockdown Sirt3 expression *in vitro*. The results showed that downregulation of Sirt3 could partially prevented the autophagy and protection induced by resveratrol after TM treatment. Our study demonstrates a pivotal role of Sirt3-mediated autophagy in mediating resveratrol-induced protection against ER stress *in vitro*, and suggests the therapeutic values of resveratrol in ER stress-associated neuronal injury conditions.

## Introduction

The endoplasmic reticulum (ER) is the most important subcellular compartment that controls protein quality and calcium storage (Hawes et al., 2015). Under stress conditions, a cellular response named ER stress is triggered to preserve ER homeostasis through initiating the unfolded protein response (UPR), or to induce cell death via activating pro-apoptotic signaling cascades (Stefani et al., 2012). Accumulating evidence supports the concept that ER stress is involved in neuronal injury in various neurological disorders, ranging from acute insults (ischemic and traumatic brain injury) to chronic degenerative diseases (Alzheimer's disease and Parkinson's disease) (Valenzuela et al., 2016).

Autophagy, a Greek word meaning self-eating, represents a self-degradative process that balances energy sources via the bulk degradation and recycling of cytosolic proteins and organelles (Balduini et al., 2009). It is a highly conserved function among eukaryotes that plays a crucial role in maintaining cell survival under both physiological and pathological conditions. However, autophagy is a double-edged sword where under certain conditions, overactivation of autophagy could disrupt cellular homeostasis and result in cell death (Thorburn, 2014). It is now accepted that ER stress is a potent trigger for autophagy, and multiple ER stress associated signaling cascades also participates in autophagy process (Yin et al., 2017). Many previous studies have highlighted the need for developing autophagy-promoting strategies for diseases that are related to ER stress (Lee et al., 2015).

Resveratrol (3,4′,5-trihydroxyl-trans-stilbene, C_14_H_12_O_3_) is a non-flavonoid polyphenolic phytoalexin discovered in the 1940s. This fat-soluble compound is present at high concentrations in grapes, peanuts, cassia and red wine (Ramprasath and Jones, 2010). Resveratrol has become a highly important natural active ingredient with many pharmacological properties, such as antioxidative, anti-inflammatory, anti-platelet, and anticancer effects (Carrizzo et al., 2013). Resveratrol is rapidly taken up after oral consumption of a low dose, and approximate 50–70% of the resveratrol could be absorbed by the body in human and rodents (Marier et al., 2002; Walle et al., 2004). In addition, resveratrol is able to cross the blood-brain barrier (BBB), and its neuroprotective activity has been demonstrated in stroke, brain trauma, seizure, and neurodegenerative diseases (Markus and Morris, 2008). However, the potential protective effects of resveratrol under ER stress conditions were not fully determined. Thus, this study was designed to investigate the effect of resveratrol in neuronal HT22 cells treated with the ER stress inducer tunicamycin (TM), and we also investigated the underlying mechanism with focus on Sirt3.

## Materials and methods

### Reagents and antibodies

TM was purchased from Sigma (St. Louis, MO, USA). Resveratrol was obtained from Calbiochem (Darmstadt, Germany). Rapamycin, chloroquine and DAPI were obtained from Tocris (Bristol, UK). Antibody against GRP78 was purchased from Bioworld (St. Louis Park, MN, USA). Antibodies against CHOP, phospho-JNK, cleaved-caspase-12 and β-actin were obtained from Santa Cruz (Santa Cruz, CA, USA). Antibodies against Beclin1, LC3, LC3II, and Sirt3 were obtained from Cell Signaling (Danvers, MA, USA).

### Cell cultures

The hippocampal neuronal HT22 cells were cultured in Dulbecco's modified Eagle's medium (DMEM) supplemented with 10% fetal bovine serum at 37°C in a 5% CO_2_ incubator.

### Cell viability assay

Cell viability was assessed by the MTT assay as described previously with minor modifications (Chen et al., 2011). Cells (1 × 10^4^ cells/well) were seeded in 96-well plates and subjected to various treatments. MTT solution at 5 mg/ml was added into each well, and incubated at 37°C in a 5% CO_2_ incubator for 4 h. After the medium was carefully removed, DMSO was added to dissolve the blue formazan product. The absorbance was detected at 490 nm.

### Lactate dehydrogenase (LDH) release assay

Cellular toxicity was determined by measuring LDH release using a LDH kit as previously described (Dai et al., 2017).

### Tunel staining

Apoptosis was detected by TUNEL staining. HT22 cells were fixed by immersing slides in 4% methanol-free formaldehyde solution in PBS for 20 min and permeabilized with 0.2% Triton X-100 for 5 min. Cells were labeled with fluorescein TUNEL reagent mixture for 60 min at 37°C and the slides were examined by fluorescence microscopy and the number of TUNEL-positive cells was counted.

### Immunocytochemistry

After being fixed with 4% paraformaldehyde for 15 min at room temperature, HT22 cells were washed with NaCl/Pi, permeabilized with 0.2% Triton X-100, and incubated with GRP78, LC3II or Sirt3 primary antibody overnight at 4°C. Cells were then incubated with Alexa 594-conjugated secondary antibody for 2 h at 37°C. Images were captured with an Olympus FV10i Confocal Microscope (Olympus, Tokyo, Japan).

### RT-PCR assays

Total RNA was prepared with the Trizol Reagent method, and 2 mg template RNA was used to synthesize the first strand of cDNA using a reverse transcription kit. The mRNA level of XBP1S was quantitated using a Bio-Rad iQ5 Gradient Real-Time PCR system (Bio-Rad Laboratories), and β-actin was used as an endogenous control.

### Enzyme activity assays

The enzymatic activity of MnSOD and catalase was measured by use of a kit (Cayman Inc.) according to the manufacturer's protocol.

### Short interfering rna (siRNA) and transfection

The specific siRNA targeted Sirt3 (sc-61556), and control siRNA (sc-37007) were purchased from Santa Cruz. The above siRNA molecules were transfected with Lipofectamine 2000 in for 48 h before various treatments.

### Western blot analysis

Proteins were loaded and separated by 10% SDS-PAGE gels, and transferred to polyvinylidene difluoride (PVDF) membranes. Membranes were blocked with 5% skimmed milk solution in TBST for 1 h, and then incubated overnight at 4°C with the primary antibodies. Immunoreactivity was detected with Super Signal West Pico Chemiluminescent Substrate (Thermo Scientific, Rockford, IL, USA).

### Statistical analysis

Statistical analysis was performed using SPSS 16.0. Statistical evaluation was performed by one-way analysis of variance (ANOVA). All samples were tested in triplicates and data from six independent experiments were used for analysis. A value of *p* < 0.05 was considered statistically significant.

## Results

### Resveratrol protects against TM-induced toxicity in HT22 cells

**Figure 1:**
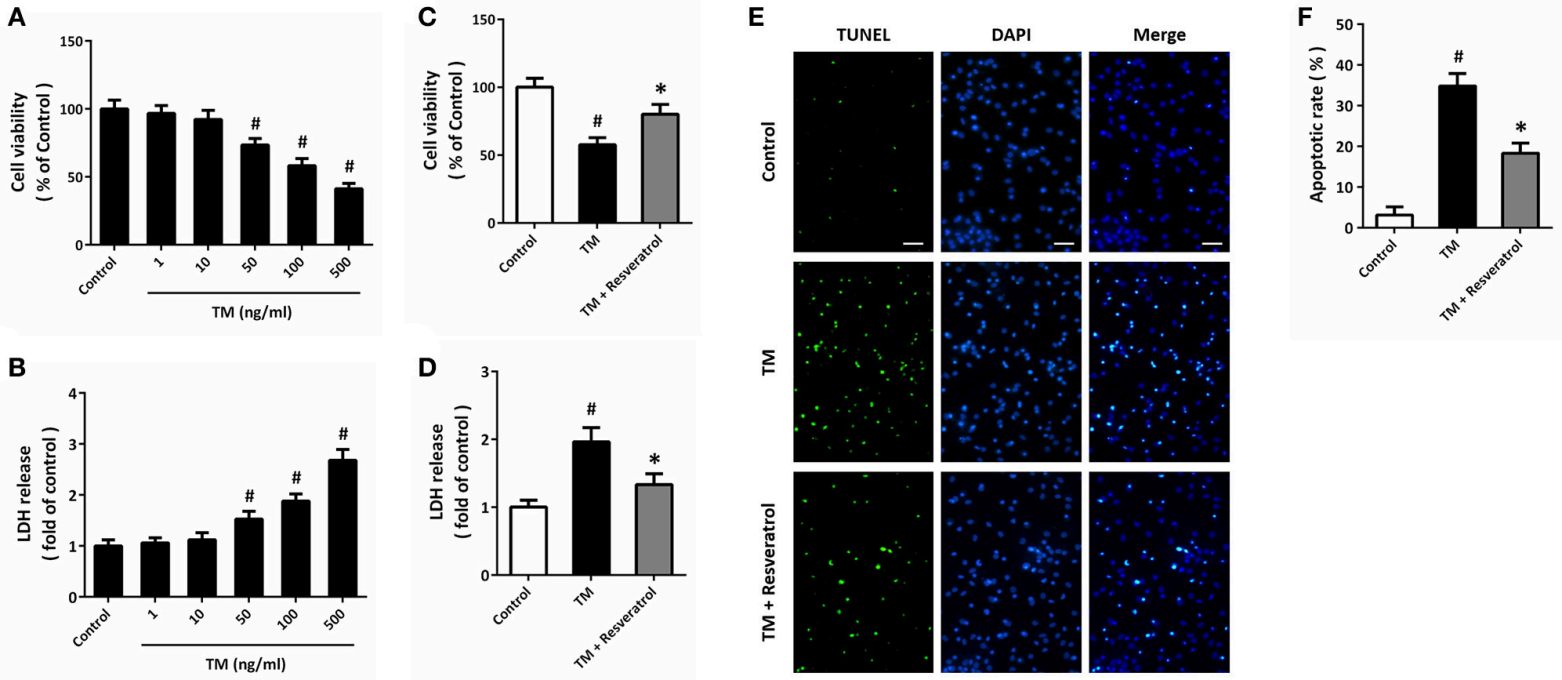
Resveratrol protects against TM-induced toxicity in HT22 cells. HT22 cells were treated with TM at different concentrations for 24 h. Cell viability was measured by MTT assay **(A)**, and cytotoxicity was determined by LDH assay **(B)**. HT22 cells were treated with 100 ng/ml TM with or without 50 μM resveratrol for 24 h, and cell viability **(C)** and LDH release **(D)** were measured. TUNEL staining was performed to detect apoptosis, and the nuclei were stained by DAPI **(E)**. The apoptotic rate was assayed **(F)**. Data are shown as mean ± SEM. ^#^*p* < 0.05 vs. Control. ^*^p < 0.05 vs. TM. Scale bar = 50 μm.

HT22 cells were treated with TM at different concentrations, and the cell viability and LDH release were measured at 24 h. The results showed that TM at 50, 100, and 500 ng/ml significantly decreased cell viability (Figure 1A) and increased LDH release (Figure 1B), while 1 and 10 ng/ml TM had no such effects. Based on these results, 100 ng/ml TM was used in the following experiments. According to previous data and our pre-experimental results (Kim et al., 2012; Luyten et al., 2017), we used 50 μM resveratrol in this study. The results showed that 50 μM resveratrol partially prevented the decrease in cell viability (Figure 1C) and increase in LDH release (Figure 1D) after TM exposure. We also detected apoptosis using TUNEL staining, and reduced number of TUNEL-positive cells was observed in resveratrol treated cells compared to that in TM-treated alone group (Figures 1E,F).

### Resveratrol attenuates TM-induced ER stress in HT22 cells

**Figure 2:**
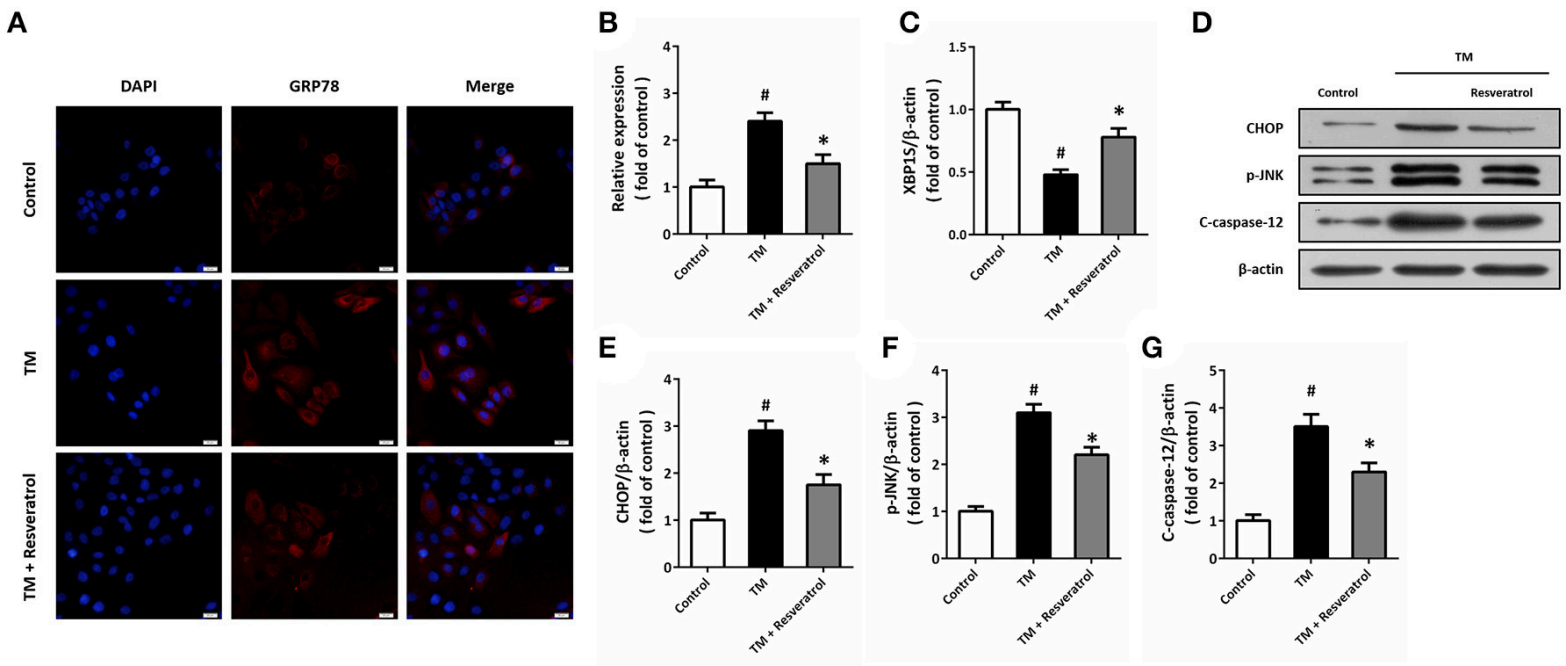
Resveratrol attenuates TM-induced ER stress in HT22 cells. HT22 cells were treated with 100 ng/ml TM with or without 50 μM resveratrol for 24 h. The expression of GRP78 protein was detected by immunofluorescence staining **(A)** and calculated **(B)**. The mRNA levels of XBP1S was examined by RT-PCR **(C)**. The expression of CHOP, p-JNK and cleaved-caspase-12 was detected by western blot **(D–G)**. Data are shown as mean ± SEM. ^#^*p* < 0.05 vs. Control. ^*^p < 0.05 vs. TM. Scale bar = 20 μm.

Immunocytochemistry was used to detect the expression of GRP78 in HT22 cells (Figure 2A), and the results showed that TM significantly increased the fluorescence of GRP78, which was partially reversed by resveratrol (Figure 2B). RT-PCR assay showed that resveratrol treatment preserved XBP1S mRNA levels after TM exposure (Figure 2C). We also detected the expression of ER stress associated pro-apoptotic factors using western blot (Figure 2D). The results showed that TM-induced CHOP induction (Figure 2E), JNK phosphorylation (Figure 2F), and caspase-12 cleavage (Figure 2G) were all significantly decreased by resveratrol, indicating the inhibition of ER stress.

### Resveratrol promotes autophagy in HT22 cells after TM exposure

**Figure 3:**
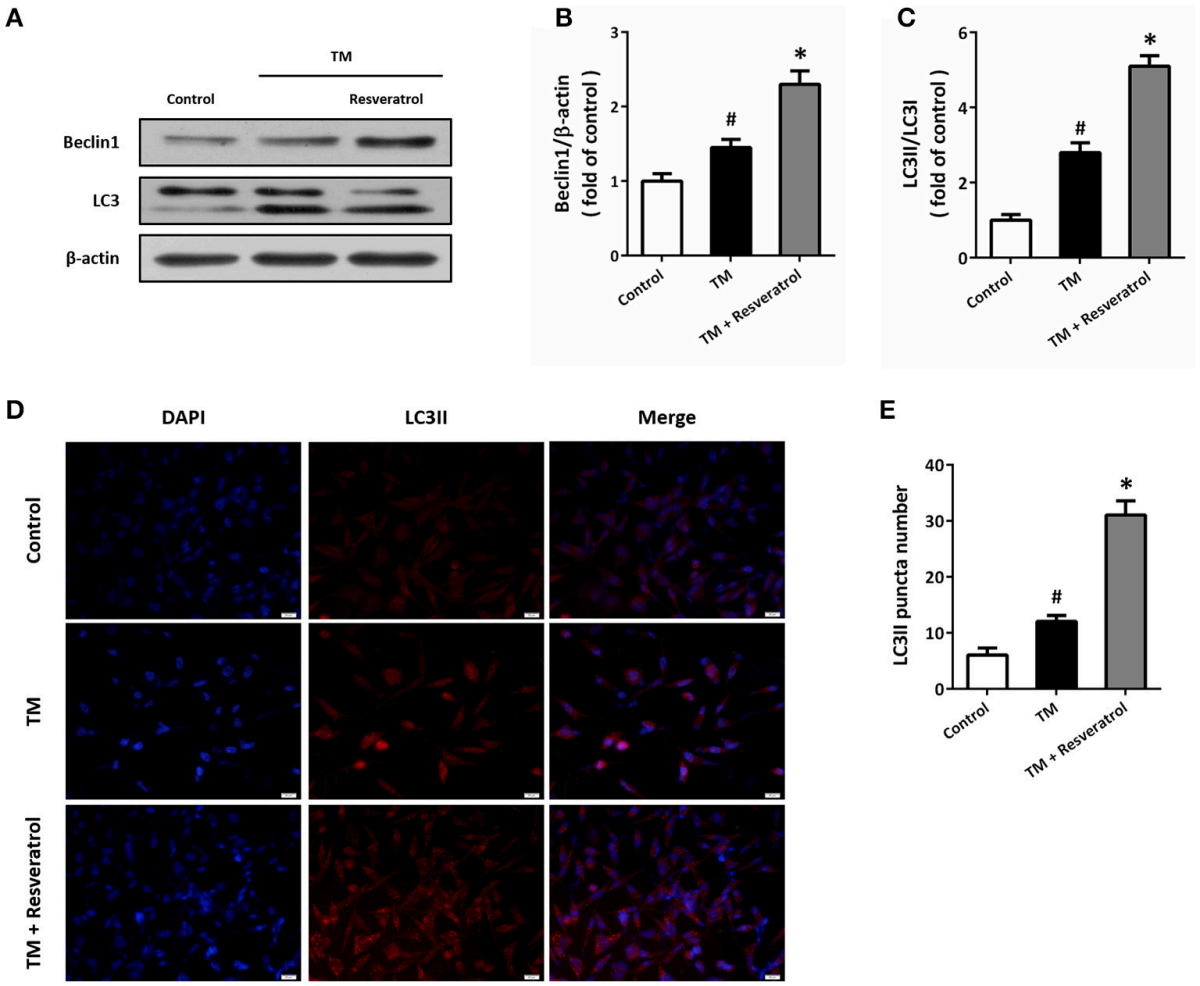
Resveratrol promotes autophagy in HT22 cells after TM exposure. HT22 cells were treated with 100 ng/ml TM with or without 50 μM resveratrol for 24 h. The expression of Beclin1 and LC3 was detected by western blot **(A–C)**. Immunofluorescence staining of LC3II was performed to assess autophagy in HT22 cells **(D)**, and the number of LC3-positve puncta was counted **(E)**. Data are shown as mean ± SEM. ^#^*p* < 0.05 vs. Control. ^*^p < 0.05 vs. TM. Scale bar = 20 μm.

Western blot was performed to detect the expression of autophagy associated proteins (Figure 3A). The results showed that TM increased the expression of Beclin1 (Figure 3B) and the ratio of LC3II/LC3I (Figure 3C), which were both further increased by resveratrol treatment. In addition, we also detected the expression of LC3II by immunocytochemistry (Figure 3D). As shown in Figure 3E, TM increased the number of LC3II puncta, and resveratrol further increased LC3II puncta number.

### Resveratrol modulates TM-induced toxicity via affecting autophagy

**Figure 4:**
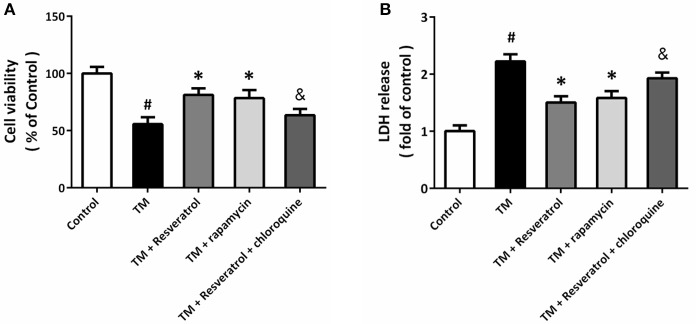
Resveratrol modulates TM-induced toxicity via affecting autophagy. HT22 cells were treated with resveratrol, rapamycin or chloroquine, and exposed to TM for 24 h. Cell viability was measured by MTT assay **(A)**, and cytotoxicity was determined by LDH assay **(B)**. Data are shown as mean ± SEM. ^#^*p* < 0.05 vs. Control. ^*^p < 0.05 vs. TM. ^&^*p* < 0.05 vs. TM + Resveratrol.

To determine the role of autophagy in resveratrol-induced protection, HT22 cells were treated with the autophagy activator rapamycin (4 μM) or the autophagy inhibitor chloroquine (10 μM). The results of MTT assay showed that rapamycin could prevent TM-induced decrease in cell viability, whereas chloroquine partially reversed resveratrol-induced protection (Figure 4A). As shown in Figure 4B, TM-induced increase in LDH release was reduced by rapamycin, and the resveratrol-induced decrease in LDH release was attenuated by chloroquine.

### Resveratrol activates sirt3 in TM-treated HT22 cells

**Figure 5:**
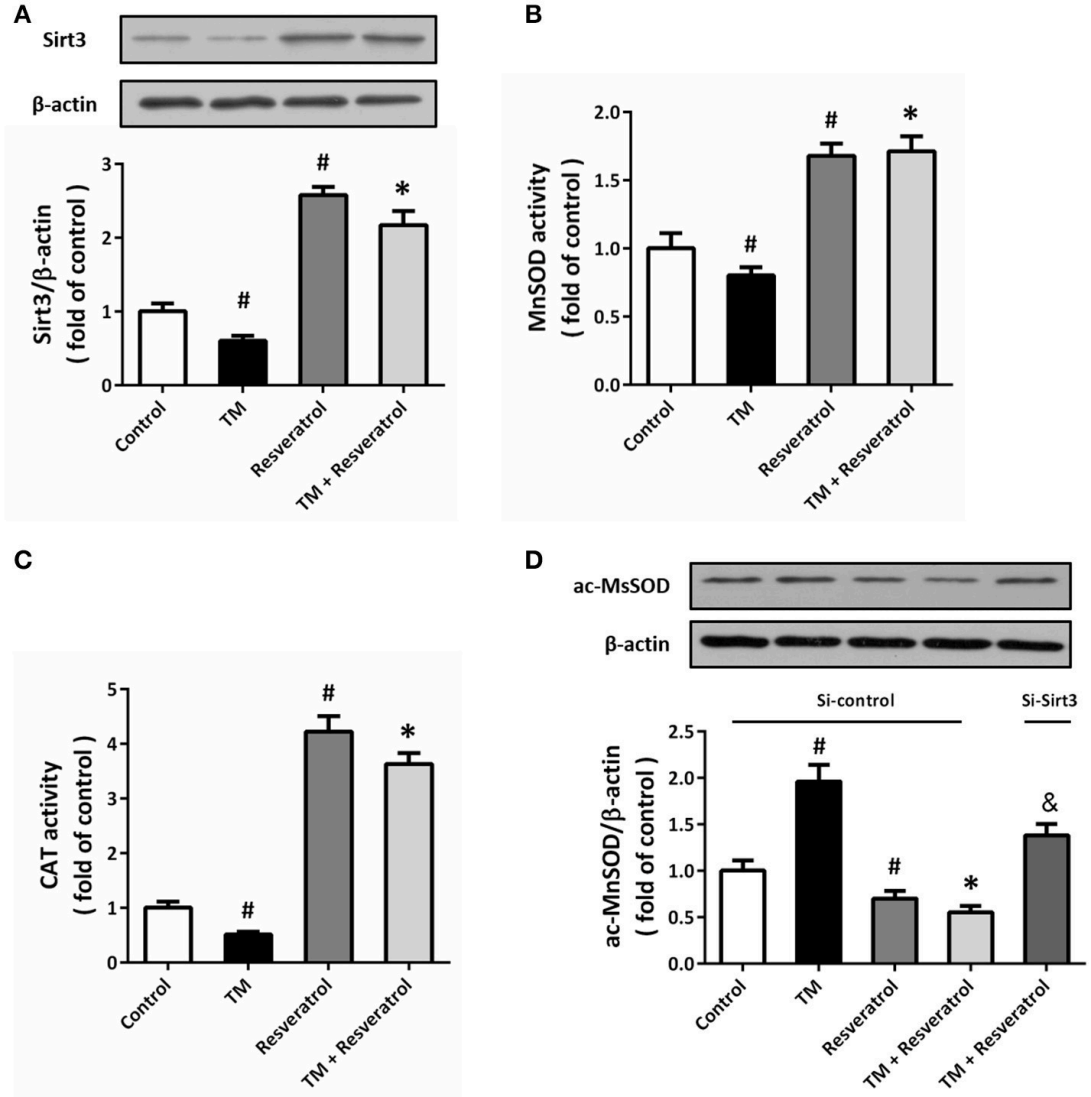
Resveratrol activates Sirt3 in TM-treated HT22 cells. HT22 cells were treated with 100 ng/ml TM with or without 50 μM resveratrol for 24 h. The expression of Sirt3 protein was detected by western blot **(A)**, and the enzymatic activities of MnSOD **(B)**, and CAT **(C)** were determined. HT22 cells were transfected with Si-control or Si-Sirt3 for 48 h, and treated with 100 ng/ml TM with or without 50 μM resveratrol for 24 h. The expression of ac-MnSOD was detected by western blot **(D)**. Data are shown as mean ± SEM. ^#^*p* < 0.05 vs. Control. ^*^p < 0.05 vs. TM. ^&^*p* < 0.05 vs. Si-control.

Western blot was performed to detect the expression of Sirt3, and the results showed that TM significantly decreased Sirt3 expression, whereas resveratrol markedly increased Sirt3 expression both in the presence and absence of TM (Figure 5A). We also measured the enzymatic activities of MnSOD and CAT, two downstream targets of Sirt3, and the results showed that resveratrol significantly increased the activities of these enzymes (Figures 5B,C). To further determine the effect of resveratrol on acetylation state of MnSOD, we detected ac-MnSOD2 expression by western blot (Figure 5D). The results showed that resveratrol inhibited MnSOD acetylation both in the presence and absence of TM, and these effects were partially prevented by Sirt3 knockdown.

### Sirt3 activation contributes to resveratrol-induced autophagy regulation

**Figure 6:**
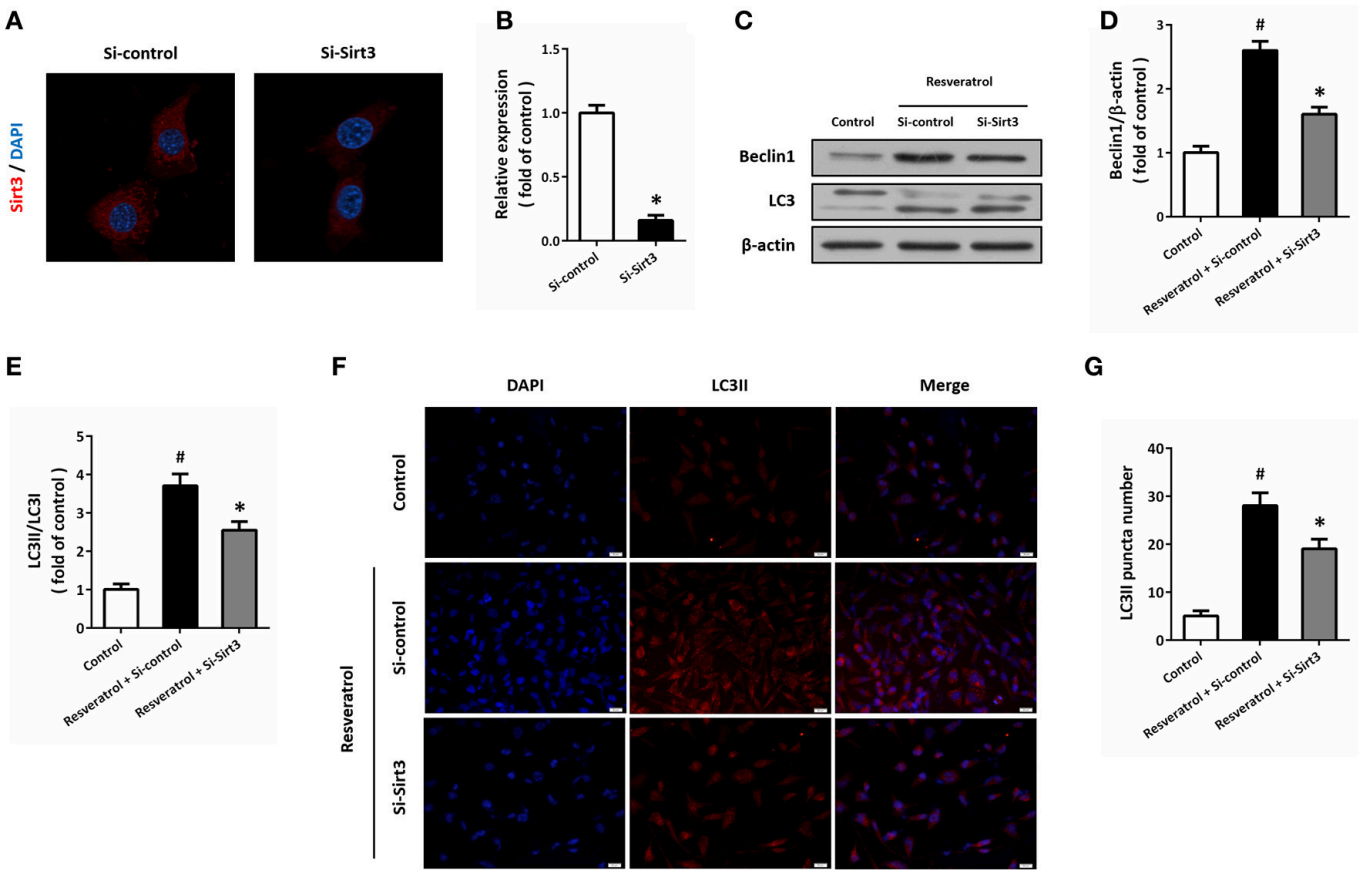
Sirt3 activation contributes to resveratrol-induced autophagy regulation. HT22 cells were transfected with Si-control or Si-Sirt3 for 48 h, and the expression of Sirt3 protein was detected by immunofluorescence staining **(A)** and calculated **(B)**. After transfection, HT22 cells were treated with resveratrol for another 24 h, and the expression of Beclin1 and LC3 was detected by western blot **(C–E)**. Immunofluorescence staining of LC3II was performed to assess autophagy in HT22 cells **(F)**, and the number of LC3-positve puncta was counted **(G)**. Data are shown as mean ± SEM. ^#^*p* < 0.05 vs. Control. ^*^p < 0.05 vs. Si-control. Scale bar = 20 μm.

HT22 cells were transfected with Si-Sirt3 to knockdown Sirt3 expression, and the results of immunocytochemistry showed that Si-Sirt3 significantly reduced Sirt3 protein levels compared to Si-control (Figures 6A,B). Downregulation of Sirt3 partially prevented the induction of Beclin1 and the increase in LC3II/LC3I ratio induced by resveratrol (Figures 6C–E). In addition, Si-Sirt3 transfection also significantly diminished the LC3II puncta in resveratrol-treated HT22 cells (Figures 6F,G).

### Knockdown of sirt3 partially prevents resveratrol-induced protection

**Figure 7:**
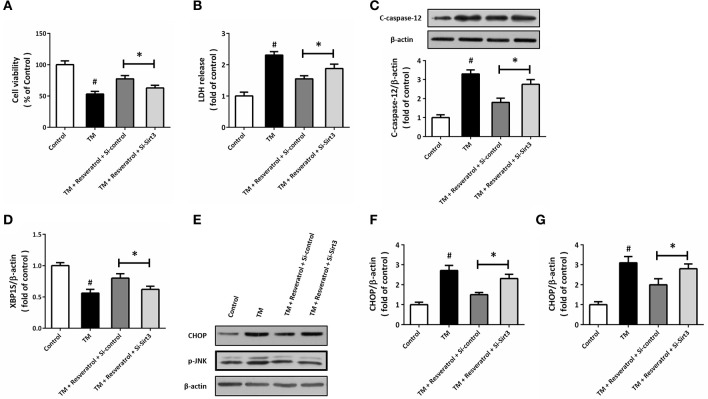
Knockdown of Sirt3 partially prevents resveratrol-induced protection. HT22 cells were transfected with Si-control or Si-Sirt3 for 48 h, and treated with TM in the presence or absence of resveratrol for another 24 h. Cell viability was measured by MTT assay **(A)**, and cytotoxicity was determined by LDH assay **(B)**. The expression of cleaved-caspase-12 was detected by western blot **(C)**. The mRNA levels of XBP1S was examined by RT-PCR **(D)**. The expression of CHOP, p-JNK, and cleaved-caspase-12 was detected by western blot **(E–G)**. Data are shown as mean ± SEM. ^#^*p* < 0.05 vs. Control. ^*^p < 0.05.

To further demonstrate the involvement of Sirt3 in resveratrol-induced protection, HT22 cells was transfected with Si-Sirt3 or Si-control before TM and resveratrol treatment. The results of MTT assay showed that resveratrol-induced increase in cell viability after TM exposure was reduced by Sirt3 knockdown (Figure 7A). In addition, the reduced LDH release induced by resveratrol was partially prevented by Si-Sirt3 transfection (Figure 7B). As shown in Figure 7C, a similar result on caspase-12 cleavage was also observed. Moreover, resveratrol-induced effects on XBP1S mRNA (Figure 7D), CHOP and p-JNK levels (Figures 7E–G) were all partially prevented by Si-Sirt3 transfection compared to Si-control.

## Discussion

Accumulating evidence has suggested that resveratrol exerts neuroprotective effects against both acute and chronic neurological disorders. The results of our study provide evidence that resveratrol attenuates ER stress associated neuronal injury via regulating autophagy in HT22 cells. We found that (a) resveratrol reduces TM-induced cell damage and apoptosis; (b) resveratrol inhibits the expression of ER related proteins; (c) resveratrol-induced promotion of autophagy contributes to its protective effects; (d) resveratrol increases Sirt3 protein expression and activity; and (e) mechanistically, knockdown of Sirt3 partially prevents the resveratrol-induced autophagy and protection.

Compared with many other natural products with neuroprotective activities, resveratrol has unique advantages. First, as a natural product, resveratrol can be easily obtained from grapes, berries, peanuts, and wines in the daily diet. In humans, resveratrol is rapidly taken up and absorbed after oral consumption, and the plasma half-life of its metabolites is approximate 9 h (Walle et al., 2004). In addition, due to its lipophilic character, tissue levels of resveratrol is much higher than those found in plasma (Timmers et al., 2012). More importantly, resveratrol can cross the BBB and act on both neurons and glial cells, making it more suitable for neurological diseases. The effects of resveratrol on ER stress was originally investigated in cancers, and resveratrol-induced induction of ER stress was found in colon and lung cancer cells (Park et al., 2007; Gu et al., 2016). However, our results showed that resveratrol significantly decreased the TM-induced activation of ER related proteins. Actually, equivalent results have also been found in acute kidney injury and non-alcoholic fatty liver diseases (Ding et al., 2017; Wang et al., 2017), and more relevantly, in Abeta25-35 treated SH-SY5Y cells (Cheng et al., 2016b). Thus, resveratrol might exert both anti-ER stress and pro-ER stress effects in different disease conditions.

Resveratrol has been previously implicated in inducing autophagy in a variety of cell types. A previous study showed that resveratrol triggered autophagic cell death through AMPK activation and JNK-dependent p62/SQSTM1 expression in chronic myelogenous leukemia cells (Puissant and Auberger, 2010). Resveratrol was shown to engage a distinct subset of LC3II and promote noncanonical autophagic degradation downstream of the Ptdlns(3)P-WIPI-Atg7-Atg5 pathway (Mauthe et al., 2011). In addition, resveratrol was also shown to induce autophagy in both glioma cells and dopaminergic SH-SY5Y cells (Yamamoto et al., 2010; Lin et al., 2014). In our *in vitro* experiments, increased number of LC3II puncta, as well as the upregulated expression of Beclin1 and LC3II, were observed after resveratrol treatment, indicating the promotion of autophagy in TM-treated conditions. In cancer cells, resveratrol caused autophagy to promote cell death, whereas increased autophagy induced by resveratrol was shown to be beneficial in many other diseases, such as spinal cord injury, brain trauma and cerebral ischemia (He et al., 2017; Zhao et al., 2017). Our results using the autophagy activator and inhibitor showed that the protective effects of resveratrol could be partially prevented by autophagy inhibition, as evidenced by both cell viability and LDH release results. Previous studies have demonstrated that resveratrol-induced autophagy is a protective mechanism in inflammatory, mitochondrial dysfunction and oxidative stress conditions (Fu, 2015; Wu et al., 2016; Zhang et al., 2017). Thus, our results suggest that resveratrol could exert protective effects against ER stress associated neurological disorders through promoting autophagy.

The sirtuins are a conserved family of NAD^+^-dependent protein deacetylases that have seven mammalian homologs (Sirtuin 1-7, aka Sirt1-7). Three sirtuin proteins, Sirt3, 4, and 5, are predominantly localized to the mitochondria, and Sirt3 has been shown to regulate almost every major aspect of mitochondrial biology in highly metabolic tissues, such as the brain (Bause and Haigis, 2013). Mitochondrial Sirt3 was demonstrated to act as a pro-survival factor playing an essential role to protect neurons under excitotoxicity (Kim et al., 2011). More recently, overexpression of Sirt3 was found to attenuate oxidative stress through regulating mitochondrial Ca^2+^ and mitochondrial biogenesis in neuronal cells (Dai et al., 2014a,b). In addition, Sirt3-mediated preservation of mitochondrial function and redox homeostasis are proved to contribute to the protective mechanism of multiple neuroprotective agents and strategies (Cheng et al., 2016a; Liu et al., 2017). Here, the results of western blot showed that resveratrol significantly increased Sirt3 protein expression both in the presence and absence of TM exposure, indicating the potential involvement of Sirt3 in its protective effects. Previous experiments have demonstrated that resveratrol could promote Sirt1 expression and activity. Resveratrol was shown to inhibit apoptosis via Sirt1 activation in hydrogen dioxide-treated osteoblast cells, and Sirt1-mediated Nrf2 activation contributed to the resistance to vascular calcification after resveratrol treatment (He et al., 2015; Zhang et al., 2016). More recently, resveratrol was shown to protect against mitochondrial Complex I deficiency via Sirt3-meidiated activation of mitochondrial SOD2 (Mathieu et al., 2016). In our study, increased activities of MnSOD and CAT were also observed in resveratrol-treated cells, and resveratrol-induced protection was partially prevented by Sirt3 knockdown using siRNA transfection. All these data indicated that the protective effects of resveratrol against TM was, at least partially, dependent on Sirt3 activation.

Acetylation and deacetylation are important posttranslational modifications employed in autophagy regulating (Bánréti et al., 2013). As class III histone deacetylases, Sirt1 and Sirt2 have been extensively implicated in modulating autophagy process (Ng and Tang, 2013). Recent studies have also identified multifaceted roles for Sirt3 in the regulation of autophagy. Sirt3 was shown to promote autophagy in AngII-induced myocardial hypertrophy through the deacetylation of FoxO1 in mice (Li et al., 2016). Sirt3 could directly bind and deacetylate SOD2, which in turn led to significant effects on mitochondrial ROS homeostasis and autophagic flux (Qiu et al., 2010; Liang et al., 2013). In our study, autophagy activation after resveratrol treatment was accompanied by increased expression of Sirt3 and elevated activity of MnSOD (also known as SOD2). More recently, Sirt3 was shown to activate autophagy and protect against ischemia in cortical neurons (Dai et al., 2017). Our results that knockdown of Sirt3 expression partially prevented the autophagy and protection induced by resveratrol, further supported the involvement of Sirt3 in autophagy regulation in neuronal cells, and indicated that resveratrol-induced protective effects were mediated by Sirt3 signaling. However, the role of Sirt3 in autophagy regulation has not been fully determined, especially some results was shown to be irreproducible (2017), and thus, some more experiments in gene knockout animals need to be performed in the future.

In summary, our results demonstrate that resveratrol exerts protective effects against TM-induced ER stress by regulating Sirt3-mediated autophagy in neuronal HT22 cells. These findings may reveal a new feature for the mechanism of resveratrol in neuroprotection, and provide further information regarding the role of Sirt3 in autophagy regulation.

## Author contributions

W-JY: designed experiments. W-JY, Y-BM, FD, and S-LD: performed experiments; Z-YH, and D-BW: prepared the manuscript; R-BL and L-KW: edited the manuscript. The entire study was supervised by W-JY.

### Conflict of interest statement

The authors declare that the research was conducted in the absence of any commercial or financial relationships that could be construed as a potential conflict of interest.
